# Descriptive study of foodborne disease using disease monitoring data in Zhejiang Province, China, 2016–2020

**DOI:** 10.1186/s12889-022-14226-1

**Published:** 2022-09-28

**Authors:** Xiaojuan Qi, Xialidan Alifu, Jiang Chen, Wenliang Luo, Jikai Wang, Yunxian Yu, Ronghua Zhang

**Affiliations:** 1 Department of Nutrition and Food Safety, Zhejiang Provincial Center for Disease Control and Prevention, 3399 Binsheng Road, Binjiang District, 310051 Hangzhou City, Zhejiang Province China; 2 Department of Epidemiology & Health Statistics, School of Public Health, School of Medicine, Zhejiang University, 310058 Hangzhou City, Zhejiang Province China; 3 Department of Public Health, Department of Anesthesiology, Second Affiliated Hospital of Zhejiang University School of Medicine, 310003 Hangzhou City, Zhejiang Province China

**Keywords:** Food safety, Microbial hazard, Foodborne pathogen, Surveillance network, China, Occupation

## Abstract

**Background:**

This study aimed to identify the epidemiology, seasonality, aetiology and clinical characteristics of sporadic foodborne diseases in Zhejiang province during 2016–2020.

**Methods:**

Descriptive statistical methods were used to analyze the data from surveillance network established by the Zhejiang Provincial Center for Disease Control and Prevention. There were 31 designated hospitals in all 11 cities which were selected using probability proportionate to size sampling method.

**Results:**

During the study period, the surveillance system received 75,124 cases with 4826 (6.42%) hospitalizations from 31 hospitals. The most common cause was Norovirus, 6120 cases (42.56%), followed by Salmonella, 3351 cases (23.30%). A significant seasonal trend was observed for the V. parahaemolyticus, with the highest rates over the summer period, peaking in August, 1171 cases (38.75%), a similar trend was also observed with Salmonella and Diarrheagenic E. coli. Norovirus infections showed the highest rate in November (904, 14.77%) and March (660,10.78%), the lowest in August, 215 cases (3.51%). Patients between 19 ~ 40 years were more likely to infected by Norovirus, V. parahaemolyticus and Diarrheagenic E. coli, patients below 1 year were the highest among patients with Salmonella infection, 881 cases (26.3%). The Norovirus, V. parahaemolyticus and Diarrheagenic E. coli infection with the highest positive detection rates among the workers were observed. The largest number cases of food categories were from aquatic product infection. The private home was the most common exposure setting.

**Conclusion:**

Our study highlighted the necessity for conducting an active, comprehensive surveillance for pathogens in all age groups, to monitor the changing dynamics in the epidemiology and aetiology of foodborne diseases to guide policies that would reduce related illnesses.

**Supplementary Information:**

The online version contains supplementary material available at 10.1186/s12889-022-14226-1.

## Introduction

Foodborne illnesses are usually infectious or virulent and caused by bacteria, viruses, parasites or chemicals that enter the body through contaminated food or water. Although, food science and related technologies are developing rapidly, but still, it remains a challenge to prevent foodborne diseases completely [1]. An estimated 600 million in the world (almost 1 in 10 people), fall ill after eating contaminated food and 420 000 die every year, resulting in the loss of 33 million healthy life years in terms of Disability Adjusted Life Years (DALYs) according to an estimate based on the 2015 data [2]. Diarrhoeal diseases account for more than 50% of foodborne diseases, according to the data released by World Health Organization (WHO), foodborne or water-borne diarrhea alone causes about 2.2 million deaths worldwide every year [3]. As in other countries, foodborne diseases characterized by acute gastrointestinal diseases are the largest food safety problem as well as the most distressing food-related threat to public health in China [4–6]. In order to reduce the disease burden, China has established a web-based foodborne disease surveillance system since 2011, which has gradually played a role in food safety incidence prevention. The surveillance contents include hygiene indicator bacteria, pathogenic bacteria, viruses, and parasites in many food categories. Moreover, sampling points are no more limited to retail and catering sites, and have been extended to processing, and sales locations.

The studies discussed the characteristics of food contamination by pathogens according to surveillance data and reflects the contamination and distribution trend of foodborne pathogens in different regions. A wide range of representative agents (including pathogenic bacteria, viruses and etc.) are covered to understand their contamination in meat and meat products [7], milk and dairy products [8], eggs and egg products [9], children’s foods [10] and ready-to-eat foods [11]. Norovirus, Salmonella spp., Vibrio parahaemolyticus (V. parahaemolyticus), Shigella and Diarrheagenic E. coli have been identified as the most common pathogens responsible for foodborne diseases in China [12, 13]. The surveillance data showed that occurrence of V. parahaemolyticus in aquatic products tended to increase over the period from 2015 to 2018 [11, 14].

Safe food supplies support national economies, trade and tourism, contribute to food and nutrition security, and underpin sustainable development. As there are a limited number of existing epidemiological studies and reports on the foodborne diseases in Zhejiang province, the need for researches has become important. The aim of this study was to summarize epidemiological characteristics of foodborne disease cases and provide effective interventions to prevent foodborne disease illnesses in Zhejiang province, we analyzed the surveillance data of foodborne disease cases caused by Norovirus, Salmonella spp., Vibrio parahaemolyticus (V. parahaemolyticus), Shigella and Diarrheagenic E. coli in Zhejiang province from 2016 to 2020.

## Methods

### Geographical position, climatic and socio-demographic feature of study site

Zhejiang Province, one of the southeastern coastal provinces of China, is located at 27°02’N to 31°11’N and 118°01’E to 123°10’E [15], the 11 cities and their subordinate counties are listed in Supplementary Table 1. Zhejiang experience a subtropical humid climate. During summer the weather is hot and humid and the temperature is around 27 to 30 °C (81 to 86 °F). During winter the temperature falls down to a minimum temperature of 2℃ to 8℃ (36 to 46 °F). Rainfall and typhoons are a common phenomenon in summers. Zhejiang province has a permanent population of 65.4 million at the end of 2021, and GDP grew 8.5% year-on-year to 7.35 trillion yuan ($1.16 trillion) in 2021 [15]. Most of Zhejiang’s wealth derives from light industry and mostly located in rural villages [16].

### Data source

Zhejiang Provincial Center for Disease Control and Prevention (ZJCDC) has collected foodborne disease relevant data through the China National Foodborne Diseases Surveillance Network (NFDSN) since 2012. 31 hospitals were inquired to detect 5 major pathogens and corresponding subtypes, including Salmonella, Norovirus, V. parahaemolyticus, Diarrheagenic E. coli and Shigella for all suspected foodborne disease cases, and reported illnesses through NFDSN since 2016. In this study the cases reported by 31 hospitals in Zhejiang province during the period 2016–2020 were included. Epidemiologists from the health departments first conducted the investigation to ascertain the full extent of the foodborne illness and the information collected for each case includes reporting region, date of occurrence, setting, etiology, food categories, number of illnesses / hospitalizations, and some other details. Unknown etiology refers to those foodborne disease cases where the confirmed etiology has not been identified. Foods was identified as the sources of disease through epidemiologic or laboratory methods and was classified into 13 categories. The food that cannot be determined was classified as “Unknown”. The GIS map data of Zhejiang Province is downloaded by the national basic geographic information center of China (http://bzdt.ch.mnr.gov.cn/).

### Statistical analysis

Total positive detection rate and hospitalization rate were calculated for each pathogen and linear trend test was used to test the change of positive detection rate and hospitalization rate annually for each pathogen. Chi-square test was used to compare the demographic characteristics, contaminated food category and food settings among four pathogens, including Salmonella, Norovirus, V. parahaemolyticus, Diarrheagenic E. coli while Shigella was not included due to limited sample sizes. Fisher exact test was used if the conditions were not met for Chi-square test. Post-hoc test was used for pairwise comparisons. Comparison was only programmed within illnesses with single etiology. Open-source software QGIS (Quantum GIS version 3.22.9) was used to map the spatial distribution of cases with positive detection rate caused by five pathogens for the period between 2016 and 2020. All statistical analyses were performed using R 3.6.2 and *P*-value was considered as significant at < 0.05.

## Results

### General epidemiological characteristics

**Fig. 1:**
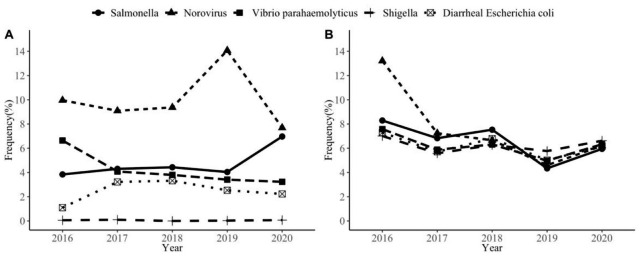
The change of positive detection rate **(A)** and hospitalization rate **(B)** of major pathogens during 2016–2020

During the study period (2016–2020), the surveillance system received 75,124 cases with 4826 (6.42%) hospitalizations from 31 hospitals. As shown in Table 1, total positive detection rate was 14,381(3.97%). The most common cause was Norovirus, 6120 cases (42.56%), followed by Salmonella, 3351 cases (23.30%), V. parahaemolyticus, 3022 cases (21.01%), Diarrheagenic E. coli,1849 cases (12.86%) and Shigella, 39 cases (0.27%). The positive detection rate increased in Salmonella and E. coli (from 3.37 to 6.59% and from 1.14 to 2.38%, respectively), while the rate for V. parahaemolyticus and Norovirus decreased during 2016–2020 (from 6.29 to 2.39% and from 10.62 to 6.62%, respectively); the rate in Shigella remained low level (Fig. 1.A). As for hospitalization rate, a significant decrease of Norovirus and Salmonella was observed during the study period as well (*P* < 0.001), with the highest in 2016 (from 12.62 to 6.55% and from 8.21 to 6.24%, respectively) (Fig. 1.B). Among all cases with positive detection, which were being hospitalized, the most common cause was Salmonella (Table 1).

**Table 1:** Percent of reported pathogens during the study period 2016–2020(N, %)

Pathogen	Positive cases	Hospitalization^#^
Norovirus	6120(42.56)	307(35.33)
Salmonella	3351(23.30)	473(54.43)
Vibrio parahaemolyticus	3022(21.01)	42(4.83)
Diarrheal Escherichia coli	1849(12.86)	36(4.14)
Shigella	39(0.27)	11(1.27)
Total	14,381	864

^#^: Hospitalization of cases with positive detection results

**Fig. 2:**
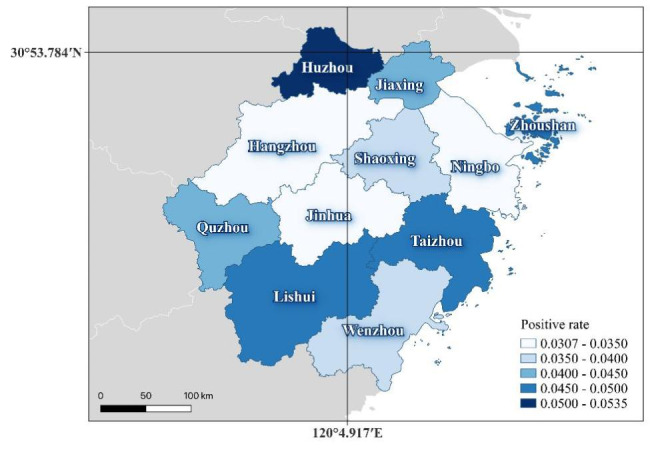
The regional distribution of cases with positive detection rate caused by five pathogens

The regional distribution of cases with positive caused by five pathogens among 11 cities, as shown in Fig. 2: 2028 cases with 5.34% detection rate in Huzhou city, 1636 (4.89%) cases in Taizhou city, 1073 (4.88%) cases in Lishui city (Fig. 2).

### Characteristics for four pathogens

For this analysis, only the highest contributing pathogens were included *(Salmonella, Norovirus, V. parahaemolyticus, and Diarrheagenic E. coli).*

### Trend and seasonality

**Fig. 3:**
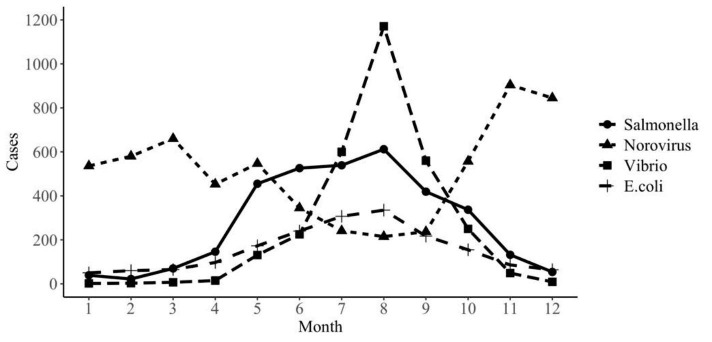
Monthly trends of selected foodborne diseases

A significant seasonal trend was observed for the V. parahaemolyticus, with the highest rates over the summer period, peaking in August, 1171 cases (38.75%). A similar trend was also observed with Salmonella and Diarrheagenic E. coli, with the peak in August, 612 cases (18.26%) and 335 cases (18.12%), respectively. Norovirus infections showed the highest rate in November (904 cases, 14.77%) and March (660 cases,10.78%) and the lowest in August, 215 cases (3.51%) (Fig. 3).

### Age, gender and occupational differences

A significant difference was observed between different age groups (*P* < 0.01), with the majority of reported cases affecting young people aged 19–40 years, as shown in Table 2. Among Salmonella infections, illnesses below one year old accounted for 26.30%, significantly higher than other three pathogens. V. parahaemolyticus showed much lower proportion for illnesses in population under 18 years old. As for gender distribution, though significantly different among four pathogens, all showed higher proportion in males (*P* < 0.05). A significant occupational difference was observed. For Norovirus, V. parahaemolyticus and Diarrheagenic E. coli infection with the highest proportion among the workers. Salmonella infections showed the highest proportion in kids living scattered,1180 cases (35.21%) (Table 2).

**Table 2:** Proportions of age composition (%), gender (%), season (%) and occupation (%) in different pathogens

Variables	Norovirus (***N =*** 6120)	Salmonella (***N =*** 3351)	V. parahaemolyticus (***N =*** 3022)	Diarrheagenic E. coli (***N =*** 1849)	***P***
**Age**					***< 0.001***
<=1	448 (7.3)	881 (26.3)	7 (0.2)	151 (8.2)	
1–3	322 (5.3)	342 (10.2)	10 (0.3)	100 (5.4)	
4–18	938 (15.3)	416 (12.4)	138 (4.6)	273 (14.8)	
19–30	1603 (26.2)	309 (9.2)	953 (31.5)	448 (24.2)	
31–40	1153 (18.8)	294 (8.8)	798 (26.4)	336 (18.2)	
41–50	585 (9.6)	244 (7.3)	430 (14.2)	199 (10.8)	
51–60	557 (9.1)	328 (9.8)	391 (12.9)	182 (9.8)	
>=60	514 (8.4)	537 (16.0)	295 (9.8)	160 (8.7)	
**Gender**					***< 0.001***
female	2650 (43.3)	1554 (46.4)	1475 (48.8)	835 (45.2)	
male	3470 (56.7)	1797 (53.6)	1547 (51.2)	1014 (54.8)	
**Season**					***< 0.001***
spring	1660 (27.1)	671 (20.0)	671 (20.0)	335 (18.1)	
summer	801 (13.1)	1677 (50.0)	1677 (50.0)	882 (47.7)	
fall	1698 (27.7)	888 (26.5)	888 (26.5)	458 (24.8)	
winter	1961 (32.0)	115 (3.4)	115 (3.4)	174 (9.4)	
**Occupation**					***< 0.001***
Farmer	969 (15.83)	760 (22.68)	643 (21.28)	344 (18.60)	
Kids living scattered	730 (11.93)	1180 (35.21)	23 (0.76)	241 (13.03)	
Worker	1003 (16.39)	254 (7.58)	735 (24.32)	381 (20.61)	
Student	913 (14.92)	240 (7.16)	154 (5.10)	229 (12.39)	
Official staff	720 (11.76)	155 (4.63)	335 (11.09)	191 (10.33)	
Unemployed	371 (6.06)	199 (5.94)	273 (9.03)	133 (7.19)	
Kids in kindergarten	216 (3.53)	223 (6.65)	2 (0.07)	83 (4.49)	
Retirees	188 (3.07)	84 (2.51)	79 (2.61)	59 (3.19)	
Others	701 (11.45)	204 (6.09)	584 (19.32)	161 (8.71)	
Unknown	309 (5.05)	52 (1.55)	194 (6.42)	27 (1.46)	

### Implicated foods and settings

**Fig. 4:**
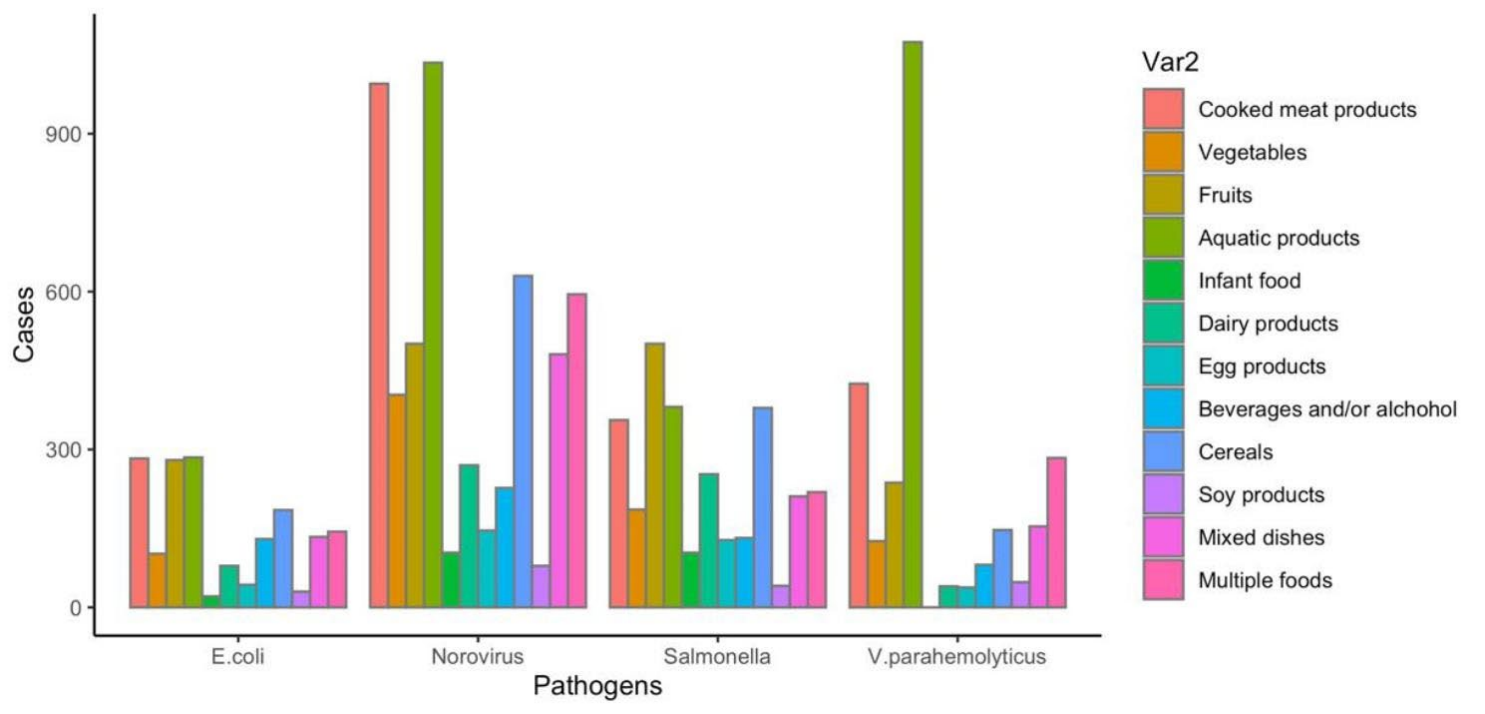
Food categories between foodborne disease cases

In this study, four type of foodborne cases were reported due to certain food vehicles, as shown in Fig. 4. Aquatic products were the most common cause for Norovirus, V. parahaemolyticus and Diarrheagenic E. coli infection (17.73%, 39.34% and 15.84%, respectively), followed by cooked meat products (17.04%, 15.57% and 15.73% respectively). The top three food vehicles in Salmonella infection were fruits (16.25%), aquatic products (12.36%) and cereals (12.29%). The places with more cases caused by four pathogens were household settings, followed by restaurants, data shown in Table 3.

**Table 3:** Reported foods (%) and settings (%) in four pathogens

Variables	Norovirus (N = 6120)	Salmonella (N = 3351)	V. parahaemolyticus (N = 3022)	Diarrheagenic E. coli (N = 1849)	***P***
**Food category**					***< 0.001***
Cooked meat products	995 (17.04)	356 (11.55)	425 (15.57)	283 (15.73)	
Vegetables	404 (6.92)	186 (6.03)	126 (4.62)	102 (5.67)	
Fruits	501 (8.58)	501 (16.25)	237 (8.68)	280 (15.56)	
Aquatic products	1035 (17.73)	381 (12.36)	1074 (39.34)	285 (15.84)	
Infant food	104 (1.78)	104 (3.37)	0 (0.00)	21 (1.17)	
Dairy products	270 (4.62)	253 (8.21)	40 (1.47)	79 (4.39)	
Egg products	146 (2.50)	128 (4.15)	38 (1.39)	43 (2.39)	
Beverages and/or alcohol	227 (3.89)	132 (4.28)	81 (2.97)	130 (7.23)	
Cereals	630 (10.79)	379 (12.29)	147 (5.38)	185 (10.28)	
Soy products	79 (1.35)	41 (1.33)	48 (1.76)	30 (1.67)	
Multiple foods	595 (10.19)	219 (7.10)	284 (10.40)	144 (8.00)	
Mixed dishes	481 (8.24)	211 (6.84)	154 (5.64)	134 (7.45)	
Unknown	372(6.37)	192(6.23)	76(2.79)	83(4.62)	
**Settings**					***< 0.001***
Restaurant	694 (11.91)	167 (5.42)	567 (20.82)	186 (10.34)	
Canteen	333 (5.71)	50 (1.62)	75 (2.75)	47 (2.61)	
Household	3260 (55.93)	2143 (69.60)	1356 (49.80)	1142 (63.52)	
Shop	167 (2.86)	84 (2.73)	99 (3.64)	91 (5.06)	
Others	1375 (23.59)	635 (20.62)	626 (22.99)	332 (18.46)	

### Symptoms

Among the Norovirus cases: 52.81% with abdominal cramps, 38.35% with vomiting, 38.28% with nausea; Salmonella caused 49.93% abdominal cramps, 28.20% fever, 19.04% nausea cases; V. parahaemolyticus caused 76.15% abdominal cramps, 46.92% nausea, 37.62% vomiting cases; Diarrheagenic E. coli caused 60.57% abdominal cramps, 25.26% nausea, 19.47% vomiting cases. Watery diarrhea was the most common symptom for four pathogens (Table 4).

**Table 4:** Reported Signs and Symptoms of patients in different pathogens

Variables	Norovirus (***N =*** 6120)	Salmonella (***N =*** 3351)	V. parahaemolyticus (***N =*** 3022)	Diarrheagenic E. coli (***N =*** 1849)	***P***
**Clinical Symptom**					
Nausea	2343 (38.28)	638 (19.04)	1418 (46.92)	467 (25.26)	**< 0.001**
Vomiting	2347 (38.35)	578 (17.25)	1137 (37.62)	360 (19.47)	**< 0.001**
Abdominal cramps	3232 (52.81)	1673 (49.93)	2301 (76.14)	1120 (60.57)	**< 0.001**
Tenesmus	30 (0.49)	23 (0.69)	12 (0.40)	12 (0.65)	0.363
Fever(>=37.5℃)	559 (9.13)	945 (28.20)	322 (10.66)	183 (9.90)	**< 0.001**
Thirsty	434 (7.09)	119 (3.55)	268 (8.87)	60 (3.24)	**< 0.001**
Fatigue	655 (10.70)	204 (6.09)	404 (13.37)	107 (5.79)	**< 0.001**
Headache	22 (0.36)	11 (0.33)	11 (0.36)	2 (0.11)	0.379
Shiver	18 (0.29)	34 (1.01)	26 (0.86)	3 (0.16)	**< 0.001**
Chest stuffiness	6 (0.10)	6 (0.18)	1 (0.03)	1 (0.05)	**0.269**
Dehydration	96 (1.57)	56 (1.67)	57 (1.89)	14 (0.76)	**0.017**
Decreased urine output	255 (4.17)	111 (3.31)	150 (4.96)	26 (1.41)	**< 0.001**
Cyanosis	1 (0.02)	2 (0.06)	1 (0.03)	0 (0.00)	0.562
Pale	25 (0.41)	14 (0.42)	33 (1.09)	4 (0.22)	**< 0.001**
Dizziness	9 (0.15)	5 (0.15)	1 (0.03)	0 (0.00)	0.164
Flushing	18 (0.29)	27 (0.81)	19 (0.63)	15 (0.81)	**0.003**
Shortness of breath	5 (0.08)	9 (0.27)	2 (0.07)	0 (0.00)	**0.015**
**Diarrhea**					**< 0.001**
Watery stool	5831 (95.78)	2951 (89.10)	2960 (98.37)	1683 (92.07)	
Rice-water stool	9 (0.15)	8 (0.24)	5 (0.17)	5 (0.27)	
Mucus stool	222 (3.65)	310 (9.36)	32 (1.06)	124 (6.78)	
Pus and blood stool	21 (0.34)	33 (1.00)	6 (0.20)	9 (0.49)	
Bloody stool	5 (0.08)	10 (0.30)	6 (0.20)	7 (0.38)	

## Discussion

Foodborne diseases impede socioeconomic development by straining health care systems, and harming national economies, tourism and trade. This study described the epidemiology of foodborne diseases caused by different pathogens in Zhejiang Province during the period 2016–2020. Over the 5 years, 75,124 cases with 4826 (6.42%) hospitalizations caused by Norovirus, Salmonella, V. parahaemolyticus, Diarrheagenic E. coli and Shigella from 31 hospitals were reported. Among 11 cities, 2028 cases in Huzhou city (14.33%), 1933 cases in Wenzhou city (13.66%), 1636 cases in Taizhou city (11.56%). The results were quite different from Sun Liang’s report, in which Wenzhou city accounts for the largest percentage of illnesses [17].

The number of illnesses caused by Norovirus ranks first among all etiologies, which is consistent with Shanghai, in which Norovirus was the most common pathogen (43.10%) [18], but quite different from the studies in China’s coastal provinces such as Hainan [19]. Wang [20] et al. reviewed 2447 papers in China that reported 1082 foodborne disease cases occurring between 1994 and 2005, in which V. parahaemolyticus caused the most events in littoral provinces, whereas in inland provinces, the largest percentage of cases were caused by Salmonella. Thus, there are regional differences in the distribution of pathogenic bacteria in China. These studies suggests that region-specific policies on foodborne disease control should be established.

Seasonality of foodborne illnesses was observed in this study. A seasonal trend was found for the V. parahaemolyticus, Salmonella and Diarrheagenic E. coli with the highest rates during summer period, peaking in August, this was similar in Enserink’s [21] and Gong’s [18] reports. However, the seasonal peak of infection attributed to some foodborne pathogens isn’t in the summer. For instance, Norovirus infections showed the highest rate in November and March and the lowest in summer, which was in line with previous studies [18, 22, 23]. Seasonality related to the temperature, humidity and rainfall, all of which may affect exposure frequency and host immune status. These findings indicated that temperature is an important factor in foodborne illnesses, and investigation of the reasons for the seasonal dominance on foodborne diseases should be the focus of surveillance.

This study showed the distinctive differences between four main pathogens with age groups. In general, the positive detection rate was higher in people aged 19 ~ 30 and 31 ~ 40 years than that in those aged < 18 and 40 + years, which were infected by Norovirus, V. parahaemolyticus and Diarrheagenic E. coli. This was partly consistent with a study in China which found incidence of foodborne diseases in youth group was higher than that in elderly group [14]. Also, a study in France which found incidence of foodborne diseases in young was higher than that in elders, in which, elders (≥ 60 years) were at least likely to get infected with V. parahaemolyticus, whereas people aged 30 ~ 44 years were the most likely get infected [24]. Similar results were observed in a Shanghai study [25]. In contrast to previous studies which found children (< 5 years) and elder people more likely to get infected with Norovirus [26, 27], our study found that the highest proportion in Norovirus infections was people aged 19–30 years old. Among Salmonella infections, cases among children aged under 1 year old accounted for 26.30%, significantly higher than other age groups. Similar findings reported in Guangdong Province that children aged < 5 years were the group most affected by Salmonella (73%), of whom the infants under 1 year old were 81.5% [28]. As for gender distribution, though significantly different among four pathogens, all showed higher proportion in male. The Norovirus, V. parahaemolyticus and Diarrheagenic E. coli infection with the highest positive detection rates in the workers were observed. Foodborne illnesses among workers are liable to occur frequently because poor hygienic conditions at workers’ camps and work situations, in the meantime, high summer temperatures impacting the transportation, distribution and storing of foods [29]. The related knowledge on what is safe should be handed down through families, work sites and credible institutions.

Analysis of exposed foods of foodborne illnesses in this study, the cases caused by Norovirus, V. parahaemolyticus and Diarrheagenic E. coli, the largest number of food categories involved were aquatic product infection (17.73%, 39.34% and 15.84%, respectively). On the contrary, a study showed the analysis of exposed foods of reported cases in Shandong Province, multiple foods (meaning more than two kinds of food) were the most commonly reported classification [30]. The reason for the different findings may be that Zhejiang is a coastal province with a vast sea area and various aquatic products. Therefore, consumers would be advised to separate raw and cooked foods, cook thoroughly as much as possible and keep food at safe temperatures to reduce the risk of foodborne diseases. However, avoiding all raw seafood should be difficult for those who are in the habit of eating seafood. As for cases infected by Salmonella, fruits, aquatic products and cooked meat products were identified as the most frequent food vehicles in the present study. Conversely, eggs have been reported as the most common classification for Salmonella infection in the US [31]. The main reason for this difference was cultural differences in eating habits. Yet it’s worth noting that, the reported classification of multiple foods relatively high as well. That means people eat more and more diverse foods, on the other hand, the category of exposed foods in national foodborne disease surveillance system is not specified in enough detail.

Analysis of the settings, according to our analysis, private home was the most common exposure setting, followed by restaurant. However, the average annual case ratios in the Republic of Korea were the highest at restaurant (57%) [32]. Among cases reported in US, restaurants also the most common settings of preparation [31]. On the contrary, Wu et al. [33] from CDC of China found that, foodborne illnesses most frequently occurred in household (32%). Similar results were observed in a EU study [34]. These findings consistent with present results, this means a large proportion of foodborne diseases caused by foods improperly prepared or mishandled at home. The effective actions can include the following aspects: know the food they use, for example, read labels on food packages, make informed choices, become familiar with common food hazards; furthermore, government should focus on home settings to reduce infections.

In regard to clinical symptoms in general, results showed similar clinical symptoms, such as nausea, abdominal pain and watery diarrhea between patients caused by four pathogens, respectively. The proportion of fever was the highest in Salmonella while lowest in Diarrheagenic E. coli. The proportion of fever in Salmonella infections in our findings was close to that in another research [35]. As Most foodborne pathogens can cause acute gastroenteritis with gastrointestinal symptoms, it is difficult to distinguish the cases infected by different pathogens by symptoms.

The limitations of this study need to be explained. First, for many reported cases, information on certain aspects, such as food category, settings and etc. were missing or incomplete, so the conclusions might not be representative of unknown classifications. Second, information and detection data were collected from 31 hospitals and several laboratories. Though detection methods were unified and regular trainings were held, there was a chance of bias caused by the different conditions and levels of hospitals and laboratories. Third, inability to conduct an epidemiological investigation due to lack of patient cooperation, there were still some missing information.

## Conclusion

Norovirus was the most common enteric pathogen detected in our surveillance during 2016–2020. Since the different epidemiological characteristics of foodborne diseases caused by different pathogens, we suggest that targeted measures be taken according to the characteristics of different etiologies and food vehicles to improve the prevention and control efficiency. The Norovirus, V. parahaemolyticus and Diarrheagenic E. coli infection with the highest positive detection rates over the workers were observed. Foodborne illnesses among workers are liable to occur frequently because hygienic conditions at workers’ camps and work situations are not always at the same standard. The related knowledge on what is safe should be handed down through families, work sites and credible institutions. Most foodborne diseases are preventable, we should further improve the identification rate of the causes of the epidemic, carry out attribution analysis for “precise prevention and control”.

## Electronic supplementary material

Below is the link to the electronic supplementary material.

Supplementary Material 1

## Data Availability

The data that support the findings of this study are available from the Foodborne Disease Case Surveillance Reporting System of the China National Center for Food Safety Risk Assessment, and these data are not publicly available. The data that support the findings of this study are available from the Foodborne Disease Case Surveillance Reporting System (https://sppt.cfsa.net.cn/goto), and these data are not publicly available.
